# Supplementary material for: Pentafluoropropionic Anhydride Derivatization and GC-MS Analysis of Histamine, Agmatine, Putrescine, and Spermidine: Effects of Solvents and Starting Column Temperature
Source: Molecules. 2023 Jan 17;28(3):939. doi: 10.3390/molecules28030939 (PMC9920471; doi:10.3390/molecules28030939)
Supplement: Supplementary file 1 [file molecules-28-00939-s001.zip › molecules-2120521-supplementary.pdf]

SUPPLEMENT

Simultaneous pentafluoropropionic anhydride derivatization and GC-MS analysis of histamine, agmatine, putrescine and spermidine: Effects of solvents and starting column temperature

Dimitrios Tsikas,[1] Bibiana Beckmann,[1] Svetlana Baskal,[1] Gorig Brunner[1]
[1]Hannover Medical School, Institute of Toxicology, Core Unit Proteomics, 30623 Hannover, Germany

Table S1. Summary of the results obtained from simultaneous GC-MS analyses in duplicate (a, b) of mixtures of putrescine, agmatine, spermidine and histamine at the indicated amounts after derivatization and extraction. The extraction solvent for the derivatives was either ethyl acetate (EA) or toluene (TOL). Selected ion monitoring of the indicated mass-to-charge ratios (*m*/*z*). The GC oven program was OTP40 (starting at 40 °C) or OTP70 (starting at 70 °C). Abbreviations: PA, peak area; PAR, peak area ratio; R, ratio; t_R, retention time. The experimental conditions are described in the Section 2.3. and the Results are reported in the Section 3.5.

PUTRESCINE *m*/*z* 340	EA		TOL		OTP40	OTP70	EA	TOL
Amount (pmol)	PA_{EA}OTP40	PA_{EA}OTP70	PA_{TOL}OTP40	PA_{TOL}OTP70	$R_{EA/TOL}$OTP40	$R_{EA/TOL}$OTP70	R_{EA}OTP40/OTP70	R_{TOL}OTP40/OTP70
60a	62534	296	63860	757	0.979	0.391	211	84
60b	62095	212	63161	570	0.983	0.371	293	111
120a	168182	614	161580	1075	1.041	0.571	274	150
120b	173450	654	168038	1012	1.032	0.646	265	166
180a	239812	721	260606	1753	0.920	0.411	333	149
180b	247399	786	253649	1797	0.975	0.437	315	141
240a	310732	930	357968	2656	0.868	0.350	334	135
240b	304258	958	337824	3287	0.901	0.291	318	103
300a	429929	1336	423857	3894	1.014	0.343	322	109
300b	406646	1166	406223	3618	1.001	0.322	349	112
Mean t_R (min)	7.945	4.035	7.942	4.027			1.96	1.97

AGMATINE *m/z* 528	EA		TOL		OTP40	OTP70	EA	TOL
Amount (pmol)	$PA_{EA}OTP40$	$PA_{EA}OTP70$	$PA_{TOL}OTP40$	$PA_{TOL}OTP70$	$R_{EA/TOL}OTP40$	$R_{EA/TOL}OTP70$	$R_{EA}OTP40/OTP70$	$R_{TOL}OTP40/OTP70$
60a	21430	33465	24432	25068	0.877	1.335	0.64	0.97
60b	21307	38847	23903	26447	0.891	1.469	0.55	0.90
120a	67974	101324	57593	69213	1.180	1.464	0.67	0.83
120b	69385	107334	60698	70173	1.143	1.530	0.65	0.86
180a	93127	140762	110015	127303	0.846	1.106	0.66	0,86
180b	100583	145011	106576	131063	0.944	1.106	0.69	0.81
240a	134427	205188	160957	184018	0.835	1.115	0.66	0.87
240b	137474	208022	149450	197940	0.920	1.051	0.66	0.76
300a	207879	324005	198588	258577	1.047	1.253	0.64	0.77
300b	195112	315898	198068	252510	0.985	1.251	0.62	0.78
Mean t_R (min)	9.262	7.260	9.263	7.262			1.28	1.28

$^{13}C_0$-SPERMIDINE *m*/*z* 361	EA		TOL		OTP40	OTP70	EA	TOL
Amount (pmol)	PA_{EA}OTP40	PA_{EA}OTP70	PA_{TOL}OTP40	PA_{TOL}OTP70	$R_{EA/TOL}$OTP40	$R_{EA/TOL}$OTP70	R_{EA}OTP40/OTP70	R_{TOL}OTP40/OTP70
60a	7011	4525	7747	7357	0.905	0.615	1.55	1.05
60b	6595	4962	7259	7281	0.909	0.681	1.33	1.00
120a	19605	11339	17655	18366	1.110	0.617	1.73	0.96
120b	18815	11672	19094	18673	0.985	0.625	1.61	1.02
180a	29583	17787	30163	29515	0.981	0.603	1.66	1.02
180b	29654	18202	29051	29211	1.021	0.623	1.63	0.99
240a	36895	22041	45810	41753	0.805	0.528	1.67	1.10
240b	35888	21505	42847	43723	0.838	0.492	1.67	0.98
300a	53987	34203	47809	50248	1.129	0.681	1.58	0.95
300b	50923	34465	47132	49597	1.080	0.695	1.48	0.95
Mean t_R (min)	11.43	9.429	11.40	9.429			1.21	1.21

$^{13}C_4$-SPERMIDINE *m/z* 365	EA		TOL		OTP40	OTP70	EA	TOL
Amount (pmol)	$PA_{EA}OTP40$	$PA_{EA}OTP70$	$PA_{TOL}OTP40$	$PA_{TOL}OTP70$	$R_{EA/TOL}OTP40$	$R_{EA/TOL}OTP70$	$R_{EA}OTP40/OTP70$	$R_{T}OTP40/OTP70$
60a	36188	22095	45576	41692	0.794	0.530	1.64	1.09
60b	34640	25702	45735	44035	0.757	0.584	1.35	1.04
120a	53067	30300	44618	45387	1.189	0.668	1.75	0.98
120b	50797	33198	46887	46051	1.083	0.721	1.53	1.02
180a	52552	32820	55979	52500	0.939	0.625	1.30	1.07
180b	53626	33625	52577	53084	1.020	0.633	1.59	0.99
240a	53619	34883	66778	60994	0.803	0.572	1.54	1.09
240b	52172	34708	62852	66840	0.830	0.519	1.50	0.94
300a	62421	41660	53896	57179	1.158	0.729	1.50	0.94
300b	58550	39960	52616	57497	1.113	0.695	1.47	0.92
Mean t_R (min)	11.42	9.42	11.42	9.42			1.21	1.21
